# RET/GFRα Signals Are Dispensable for Thymic T Cell Development *In Vivo*

**DOI:** 10.1371/journal.pone.0052949

**Published:** 2012-12-27

**Authors:** Afonso Rocha Martins Almeida, Sílvia Arroz-Madeira, Diogo Fonseca-Pereira, Hélder Ribeiro, Reena Lasrado, Vassilis Pachnis, Henrique Veiga-Fernandes

**Affiliations:** 1 Instituto de Medicina Molecular, Faculdade de Medicina de Lisboa, Lisboa, Portugal; 2 Division of Molecular Neurobiology, Medical Research Council (MRC), National Institute for Medical Research, London, United Kingdom; Institut Pasteur, France

## Abstract

Identification of thymocyte regulators is a central issue in T cell biology. Interestingly, growing evidence indicates that common key molecules control neuronal and immune cell functions. The neurotrophic factor receptor RET mediates critical functions in foetal hematopoietic subsets, thus raising the possibility that RET-related molecules may also control T cell development. We show that *Ret*, *Gfra1* and *Gfra2* are abundantly expressed by foetal and adult immature DN thymocytes. Despite the developmentally regulated expression of these genes, analysis of foetal thymi from *Gfra1*, *Gfra2* or *Ret* deficient embryos revealed that these molecules are dispensable for foetal T cell development. Furthermore, analysis of RET gain of function and *Ret* conditional knockout mice showed that RET is also unnecessary for adult thymopoiesis. Finally, competitive thymic reconstitution assays indicated that *Ret* deficient thymocytes maintained their differentiation fitness even in stringent developmental conditions. Thus, our data demonstrate that RET/GFRα signals are dispensable for thymic T cell development *in vivo*, indicating that pharmacological targeting of RET signalling in tumours is not likely to result in T cell production failure.

## Introduction

T cell development occurs mainly in the thymus [1]. However, by the time T cell precursors reach this primary lymphoid organ, they are not fully committed, and only later receive the cues that engage them on a T cell fate [1], [2], [3]. Thus, the thymic microenvironment is thought to provide appropriate signals that maintain a balance between thymocyte selection, proliferation and cell death [4], [5]. These signals are dependent on thymocyte receptors and their cognate ligands, either soluble or membrane bound, which are obtained from the thymic microenvironment. Determinant factors to T cell precursor development have a mesenchymal or hematopoietic cell origin and are believed to trigger a gene expression program leading to specific cell fates [1], [2], [3]. Among major known molecular players in T cell development are Notch-Delta and TCR-MHC interactions [6], [7]. However, identification of additional regulators of thymocyte development is still an unmet need in T cell biology.

Although recent advances have added into the complexity of T cell developmental stages, the latter can still be defined based on the expression of the T cell receptor (TCR) and the co-receptors CD4 and CD8 [2], [4], [8]. Initially, immature (CD3^−^) thymocytes are double-negative (DN) CD4^−^CD8^−^, then develop into double-positive (DP) CD4^+^CD8^+^ thymocytes through an immature CD8^+^CD3^−^ (ImmCD8) intermediate stage, and ultimately are selected into CD4^+^CD3^+^ or CD8^+^CD3^+^ mature compartments [2], [8].

T cell development starts in embryonic life [4], [9]. Seeding of the embryonic thymus occurs around E13.5 and few thymocytes are beyond DN stage until E16.5 [4]. Full maturation of αβ T cells is residual before E19.5, but some unique γδ T cell populations are produced exclusively at defined foetal stages [2], [4].

Previous studies showed expression of neurotrophic factors of the glial cell-line derived neurotrophic factor (GDNF) family (GFLs) in the thymus [10], [11]. Productive signalling by GFLs is dependent on their association to a co-receptor (GFRα1 to 4), which also confers a degree of specificity to each GFL. Thus, GFRα1 is required to GDNF signalling, GFRα2 to NRTN, GFRα3 to ARTN and GFRα4 to PSPN [12]. GFRα molecules cooperate mainly with the transmembrane tyrosine kinase receptor RET for downstream signalling [12].

Activating mutations of *Ret* have been linked to cancer, i.e., somatic chromosomal rearrangements result in Papillary Thyroid Carcinoma, point mutations of RET lead to Multiple Endocrine Neoplasia 2 syndrome and RET is also differentially expressed in acute myeloid leukaemia [13], [14]. Thus, RET inhibitors were recently developed for specific human cancer therapies [15], [16].

RET signalling axes are critical to the neuronal system and kidney [12], but recent evidence indicates that RET signals are also key to intestinal lymphoid organ development [17], [18]. Interestingly, it was shown that RET is expressed by mature lymphocytes [19] and GDNF promotes DN thymocytes survival *in vitro* [11]; thus, raising the exciting possibility that RET signalling may control thymocyte development *in vivo*.

In this study, we used cellular, molecular and genetic approaches to investigate the role of RET in foetal and adult thymic T cell development *in vivo*. We show that *Ret*, *Gfra1* and *Gfra2* are abundantly expressed in developing thymocytes, particularly in the earliest DN stages. Despite the developmentally regulated expression of these genes, analysis of E18.5 thymi from *Ret^−/−^*, *Gfra1^−/−^* or *Gfra2^−/^* ^−^ embryos revealed an insignificant impact of these molecules in T cell development. Sequentially, we used *Re*t conditional knockout mice in order to ablate *Ret* expression in T cell development. Similarly to foetal life, we found that RET is dispensable to thymocyte development in adulthood. This conclusion was further supported by the fact that RET gain of function mutations did not alter thymocyte differentiation. Finally, we employed competitive reconstitution chimeras to uncover subtle effects of *Ret* deficiency within the thymus. This very sensitive method revealed that the competitive fitness of developing *Ret* deficient thymocytes was intact. Thus, our data demonstrate that RET signalling is dispensable to thymic T cell development *in vivo*.

## Results

### *Ret*, *Gfra1*, *Gfra2*, *Gdnf* and *Nrtn* are expressed in the foetal thymus

**Figure 1:**
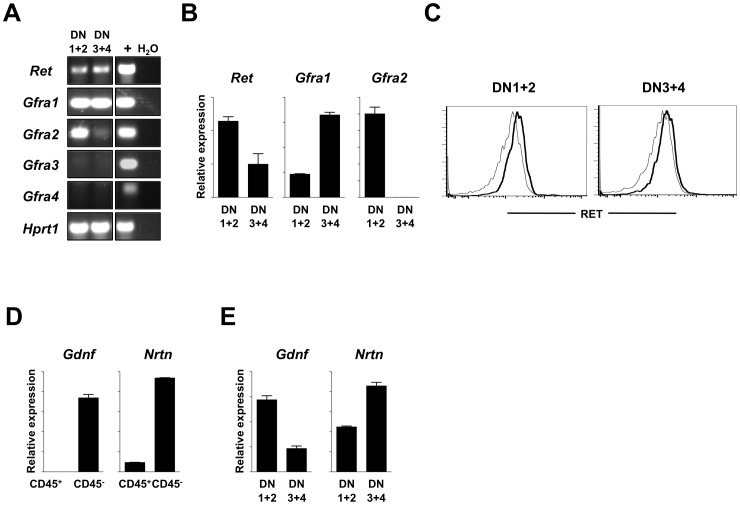
Expression of *Ret* and its signalling partners in foetal thymic populations. **A.** E15.5 DN1–2 and DN3–4 thymocytes were purified by flow cytometry. Results show RT-PCR analysis. **B.** E15.5 DN1–2 and DN3–4 thymocytes were purified by flow cytometry. Results show quantitative RT-PCR normalized to *Hprt1*. Error bars show s.e. Results from three independent measurements are represented. **C.** RET expression in DN1–2 and DN3–4 thymocytes was determined by flow cytometry. RET: black bold line; Isotype control: grey line. **D.** Thymic E15.5 CD45^+^ or CD45^−^ cells were purified by flow cytometry. Quantitative RT-PCR analysis was normalized to *Hprt1*. Error bars show s.e.. Results from three independent measurements are represented. **E.** DN1–2 and DN3–4 thymocytes were obtained and analyzed as in Figure 1B.

Previous reports have shown the expression of *Ret*, *Gfra1* and *Gdnf* in the thymus [10], [11]. Initially we investigated the expression of *Ret* and its co-receptors in E15.5 thymocyte subsets by RT-PCR. Although most E15.5 thymocytes are at the DN stage [4], due to minute cell numbers available at this developmental stage we sorted DN1+DN2 (pooling CD4^−^CD8^−^CD3^−^CD44^+^CD25^−^ and CD4^−^CD8^−^CD3^−^CD44^+^CD25^+^ cells) and DN3+DN4 thymocytes (CD4^−^CD8^−^CD3^−^CD44^−^CD25^+^ and CD4^−^CD8^−^CD3^−^CD44^−^CD25^−^) by flow cytometry. We found that while *Ret*, *Gfra1* and *Gfra2* were expressed in the foetal thymus, *Gfra3* and *Gfra4* were absent (Fig. 1A). Sequentially, quantitative RT-PCR analysis confirmed expression of *Ret* and *Gfra1* in thymocytes at all DN developmental stages, a finding also confirmed at the protein level for RET (Fig. 1B, 1C). In contrast, *Gfra2* was present in DN1+DN2 but absent from later DN stages (Fig. 1B). Sequentially, we evaluated the expression of the RET-ligands *Gdnf* and *Nrtn* in the thymic environment. We found that the main source of these transcripts were CD45^−^ cells (Fig. 1D), while hematopoietic (CD45^+^) DN thymocytes only expressed minute levels of *Gdnf* and *Nrtn* (Fig. 1D, 1E). Thus, we confirmed that the molecules required for active RET signalling are expressed in the embryonic thymus, suggesting a role for these neurotrophic factor signalling axes in the early stages of foetal thymocyte development.

### RET, GFRα1 and GFRα2 are dispensable for foetal thymocyte development

In order to determine whether RET mediated signals are required for foetal thymocyte development, we analyzed E18.5 thymus from *Ret^−/−^*, *Gfra1^−/−^* or *Gfra2^−/−^* animals [20], [21], [22], thus including in our analysis DN thymocytes and emergent immCD8, DP and γδ TCR thymocytes.

**Figure 2:**
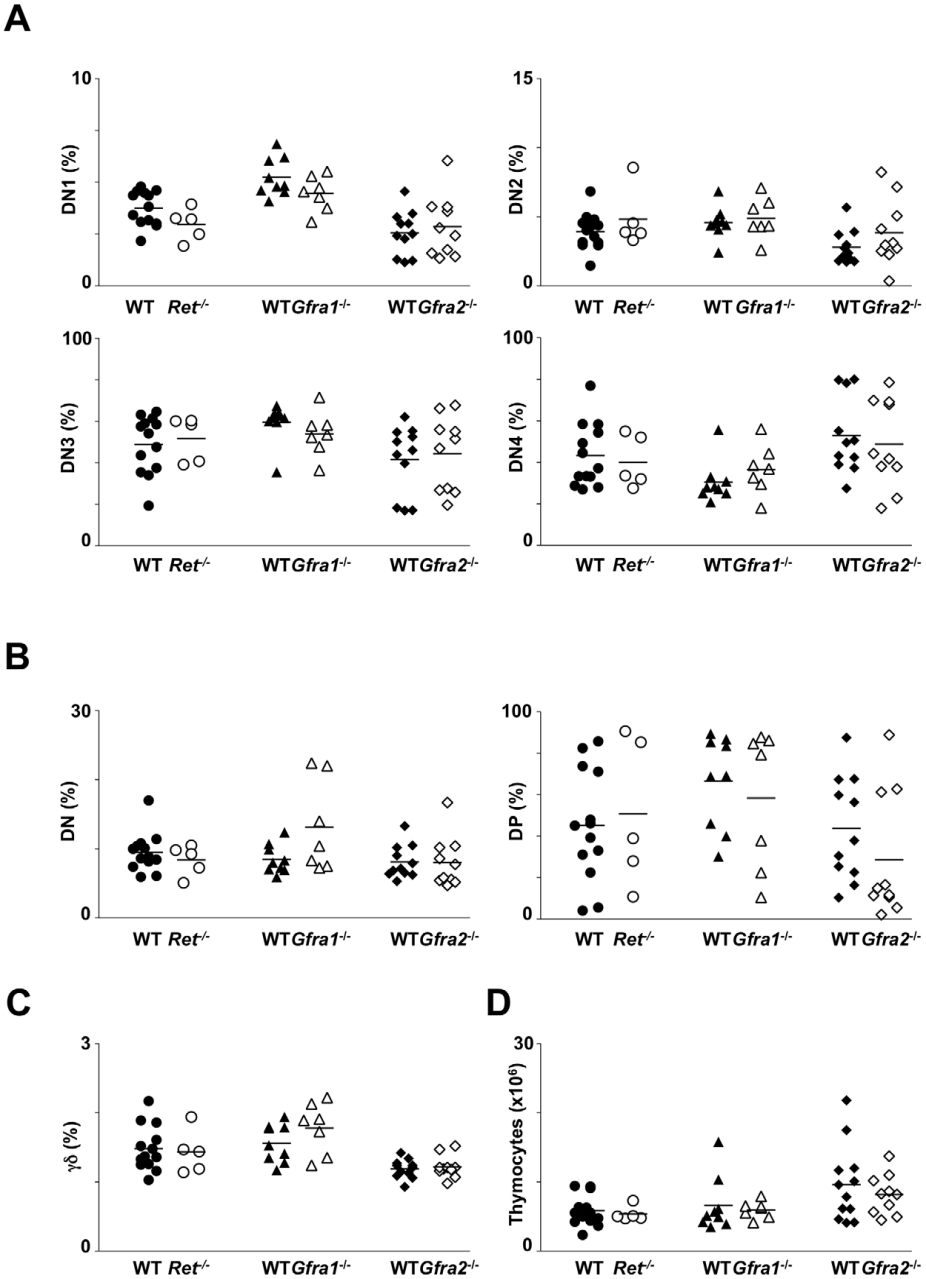
Impact of *Ret*, *Gfra1* or *Gfra2* ablation in embryonic thymocyte development. E18.5 thymocytes were analyzed by flow cytometry. **A.** DN thymocytes were gated on CD45^+^Lin^−^CD3^−^CD4^−^CD8^−^ cells. Results show percentage of DN1–DN4 in *Ret*, *Gfra1*and *Gfra2* deficient mice. Null mice: open symbols; WT littermate controls: full symbols; Mean value: dash line. **B.** Percentage of DN and DP thymocytes gated on CD45^+^Lin^−^γδTCR^−^ analyzed as in Figure 2A. **C.** Percentage of γδ TCR expressing thymocytes analyzed as in Figure 2A. **D.** Absolute number of total thymocytes in *Ret*, *Gfra1*and *Gfra2* deficient mice analyzed as in Figure 2A. Two-tailed student *t*-test analysis was performed between knockouts and respective WT littermate controls. No statistically significant differences were found.

Since expression of *Ret*, *Gfra1* and *Gfra2* is higher in early DN thymocytes (DN1 and DN2) (Fig. 1B), we initially evaluated these differentiation stages in *Ret*, *Gfra1* or *Gfra2* deficient embryos. We found that both the percentage and cell number of DN1–4 subsets were similar between *Ret*, *Gfra1* or *Gfra2* deficient embryos and their respective WT littermate controls (Fig. 2A; Fig. S1). Similarly, we found that total DN and ImmCD8 were equally represented in mutant embryos and their WT controls (Fig. 2B; Fig. S1).

Sequentially, we analyzed later stages of the αβ TCR lineage development. Absolute numbers of DP thymocytes from *Ret* ^−/−^, *Gfra1^−/−^* or *Gfra2^−/−^* embryos were identical to WT littermate controls (Fig. 2B; Fig. S1). Similarly, the fraction and absolute numbers of γδ TCR thymocytes, which are the majority of CD3^+^ cells at E18.5 [4], were unperturbed in *Ret*, *Gfra1* or *Gfra2* deficient animals (Fig. 2C; Fig. S1). Consequently, absolute numbers of total thymocytes from *Ret*, *Gfra1* or *Gfra2* deficient embryos were similar to their WT littermate controls (Fig. 2D). Thus, we conclude that signals mediated by RET or by its co-receptors GFRα1 or GFRα2 are not required for foetal thymocyte development *in vivo*.

### RET and its co-receptors are expressed in adult thymocytes

The thymic environment supports T cell development in embryonic and adult life. Nevertheless, T cell development in the foetus and adult thymus employs differential pathways, leading to different viability, proliferation and lineage commitment [4]. Thus, we investigated whether *Ret* related genes maintain their expression through adult thymopoiesis.

**Figure 3:**
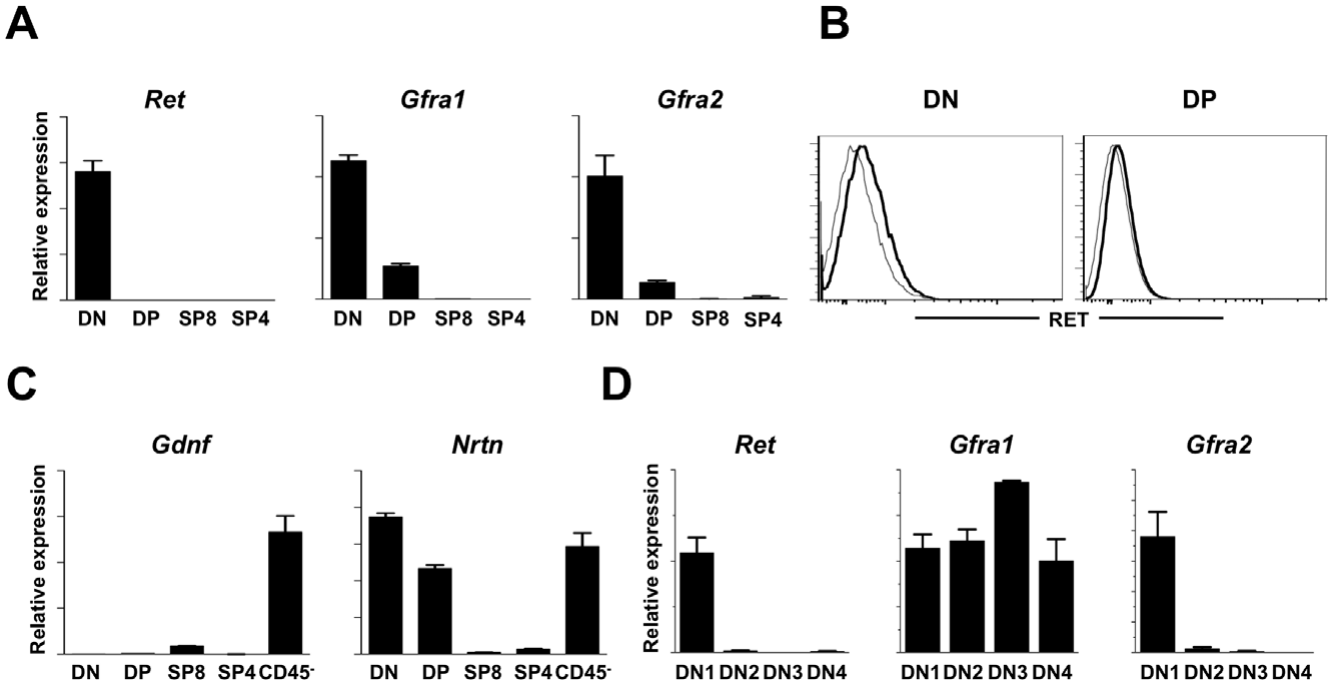
Expression of *Ret* and its signalling partners in adult thymic populations. **A.** DN, DP, SPCD8^+^ and SPCD4^+^ thymocytes were purified by flow cytometry. Results show quantitative RT-PCR normalized to *Hprt1*. Error bars show s.e.. Results from three independent measurements are represented. **B.** RET expression in DN and DP thymocytes was determined by flow cytometry. RET: black bold line; Isotype control: grey line. **C.** Thymic DN, DP, SPCD8^+^ and SPCD4^+^ thymocytes and CD45^−^ cells were purified by flow cytometry. Quantitative RT-PCR analysis was normalized to *Hprt1*. Error bars show s.e.. Results from three independent measurements are represented. **D.** DN1–2 and DN3–4 thymocytes were purified by flow cytometry. Results show quantitative RT-PCR normalized to *Hprt1*. Error bars show s.e.. Results from three independent measurements are represented.

DN (CD4^−^CD8^−^CD3^−^), DP, single-positive CD4^+^ T cells (SPCD4) and single positive CD8^+^ T cells (SPCD8) were FACS sorted and analyzed by quantitative RT-PCR analysis. RT-PCR analysis revealed that similarly to the foetal thymus only *Ret* and its co-receptors *Gfra1* and *Gfra2* were expressed in the adult thymus (Fig. S2). Quantitative RT-PCR confirmed that *Ret*, *Gfra1* and *Gfra2* expression was mainly expressed by DN thymocytes, although low levels of *Gfra1* and *Gfra2* expression were also expressed by DP thymocytes, a finding also confirmed at the protein level for RET (Fig. 3A, 3B). Sequentially, we evaluated the expression of the RET-ligands *Gdnf* and *Nrtn* in the adult thymus. While *Gdnf* expression was mostly found on CD45^−^ cells, *Nrtn* was expressed both by CD45^−^ and CD45^+^ DN and DP thymocytes (Fig. 3C).

Dissection of DN cells into DN1-DN4 subsets further revealed that DN1 thymocytes were the only DN subset that co-expressed appreciable levels of *Ret*, *Gfra1* and *Gfra2*, while all other DN subsets expressed *Gfra1* but only minute levels of *Ret* (Fig. 3D). Thus, we conclude that the expression of RET signalling partners in adult thymocytes mirrors to large extend the expression patterns of foetal thymocytes, ie, *Ret*, *Gfra1*and *Gfra2* are most abundant in the earliest stages of T cell development, while *Gdnf* and *Nrtn* are mainly produced by non-hematopoietic thymic cells.

### RET-mediated signals are dispensable for adult T cell development

**Figure 4:**
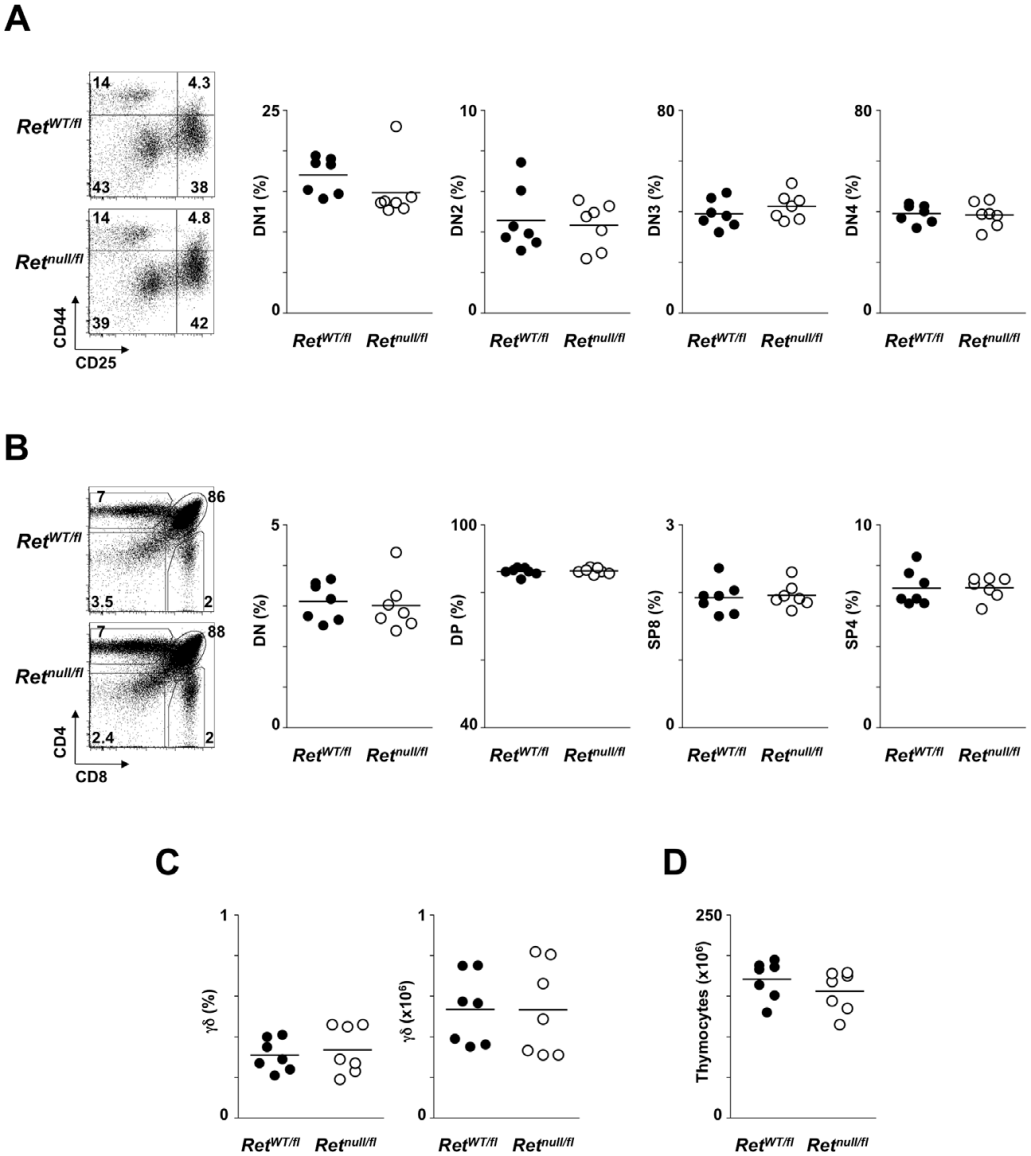
Impact of *Ret* ablation in adult thymic development. 8 week old *Ret* conditional knockout *hCD2*Cre^/^ *Ret^null/fl^* and control *hCD2*Cre^−^/*Ret^wt/fl^* mice were analyzed by flow cytometry. **A.** Left: representative flow cytometry analysis of CD4^−^CD8^−^CD3^−^ thymocytes. Percentages are indicated. Right: Results show percentage of DN1-DN4 in *hCD2*Cre/*Ret^null/fl^* (open circle) and control *hCD2*Cre^−^/*Ret^wt/fl^* (full circle) mice. Mean value: dash line. **B.** Left: representative flow cytometry analysis of CD4 versus CD8 expression profile. Percentages are indicated. Right: Results show percentage of DN, DP, SP4 and SP4 in *hCD2*Cre/*Ret^null/fl^* (open circle) and control *hCD2*Cre^−^/*Ret^wt/fl^* (full circle) mice. Mean value: dash line. **C.** Proportion and absolute numbers of γδ TCR expressing thymocytes in *hCD2*Cre/*Ret^null/fl^* (open circle) and control *hCD2*Cre^−^/*Ret^wt/fl^* (full circle) mice. Mean value: dash line. **D.** Absolute thymocyte numbers. Two-tailed student *t*-test analysis was performed between mutant and respective control mice. No statistically significant differences were found.

*Ret^−/−^* animals die perinatally due to kidney failure, hindering analysis of adult T cell development [22]. Thus, in order to determine the role of RET signalling in adult thymopoiesis, we developed a *Ret* conditional knockout model (*Ret* ^fl/fl^) that allows a lineage targeted strategy for *Ret* ablation. These mice were bred to human CD2-Cre animals that ensure Cre activity from DN1 stage onwards [23] (Fig. S2). Analysis of the offspring of this breeding at 8 weeks of age showed that despite a marginal reduction in DN1 thymocyte numbers in *CD2*Cre/*Ret* ^null/fl^ animals, the subsequent DN stages were similarly represented in *CD2*Cre/*Ret* ^null/fl^ and *CD2*Cre/*Ret* ^WT/fl^ mice (Fig. 4A; Fig. S3). Analysis of DN to SP αβ T cell development showed similar fractions and absolute numbers of thymocytes within each subset (DN, DP, SP4 and SP8) in both *CD2*Cre/*Ret* ^null/fl^ and *CD2*Cre/*Ret* ^WT/fl^ mice (Fig. 4B; Fig. S3). Finally, the fraction and absolute numbers of thymic γδ TCR expressing T cells and total thymocyte numbers were not affected by *Ret* deletion (Fig. 4C, 4D). Thus, altogether our data indicate that RET-mediated signals are dispensable for foetal and adult thymic T cell development *in vivo*.

### *Ret* gain-of-function mutations do not affect thymopoiesis

Over expression and expression of gain-of-function forms of RET are characteristic of proliferative cell phenotypes and oncogenic diseases [13]. Thus, in order to elucidate whether improved RET signals could affect thymopoiesis, we used a genetic model that drives a constitutively activated form of RET in *Ret* expressing cells (*Ret* ^MEN2B^) [24]. These mice harbour a single point mutation (Met919Thr) introduced into the endogenous *Ret* gene locus, thus resulting in improved ligand-dependent RET activation [24].

**Figure 5:**
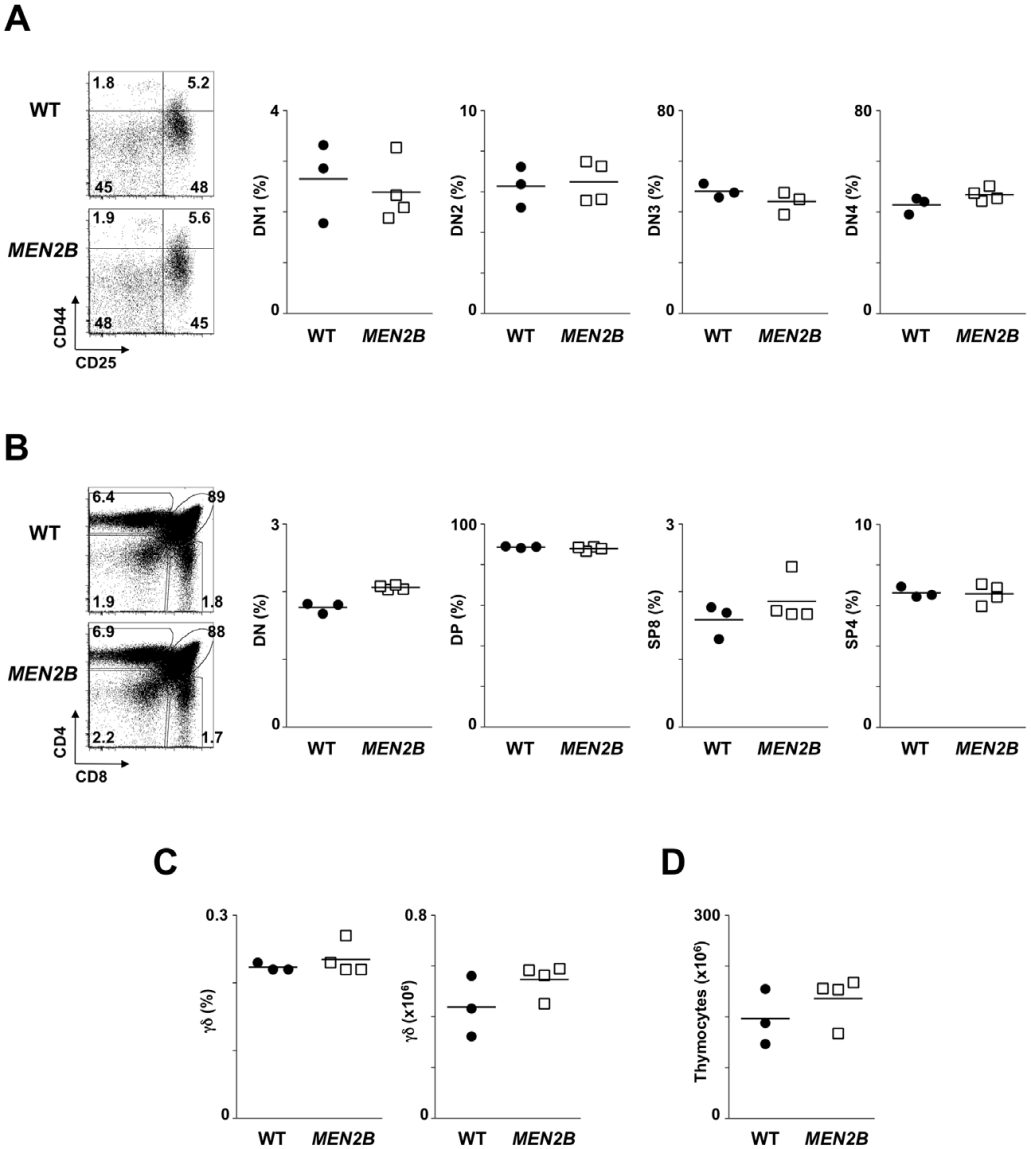
*Ret* ^MEN2B^ gain-of-function mutation in adult thymocyte development. 8 week old *Ret* ^MEN2B/MEN2B^ (*MEN2B*) and their WT littermate controls were analyzed by flow cytometry. **A.** Left: representative flow cytometry analysis of CD4^−^CD8^−^CD3^−^ thymocytes. Percentages are indicated. Right: Results show percentage of DN1–DN4 in *MEN2B* (open squares) and WT control (full circle) mice. Mean value: dash line. **B.** Left: representative flow cytromety analysis of CD4 versus CD8 expression profile. Percentages are indicated. Right: Results show percentage of DN, DP, SP4 and SP4 in in *MEN2B* (open squares) and WT control (full circle) mice. Mean value: dash line. **C.** Proportion and absolute numbers of γδ TCR expressing thymocytes in *MEN2B* (open squares) and WT control (full circle) mice. Mean value: dash line. **D.** Absolute thymocyte numbers. Two-tailed student *t*-test analysis was performed between knockouts and respective controls. No statistically significant differences were found.

Analysis of *Ret* ^MEN2B/MEN2B^ and their WT littermate controls at 8 weeks of age revealed that DN (DN1–DN4) to SP mature αβ T cell development had similar fractions and absolute numbers (Fig. 5A–C; Fig. S4). Consequently, total thymocyte numbers were not affected by the *Ret* ^MEN2B^ gain-of-function mutation (Fig. 5D), demonstrating that this *Ret* gain-of-function mutation does not affect thymopoiesis.

### RET signalling is dispensable for thymic reconstitution

*Ret* signalling has been implicated in the survival of a variety of peripheral and central neural cells, including motor, sensory and autonomic neurons [12]. Furthermore, it was shown that the RET ligand GDNF could promote thymocyte survival in foetal thymus organ culture (FTOC) systems [11]. Thus, it is plausible that subtle effects of RET signalling on thymocyte development might be masked in *Ret* conditional knockouts by compensatory mechanisms at later stages of thymocyte differentiation.

**Figure 6:**
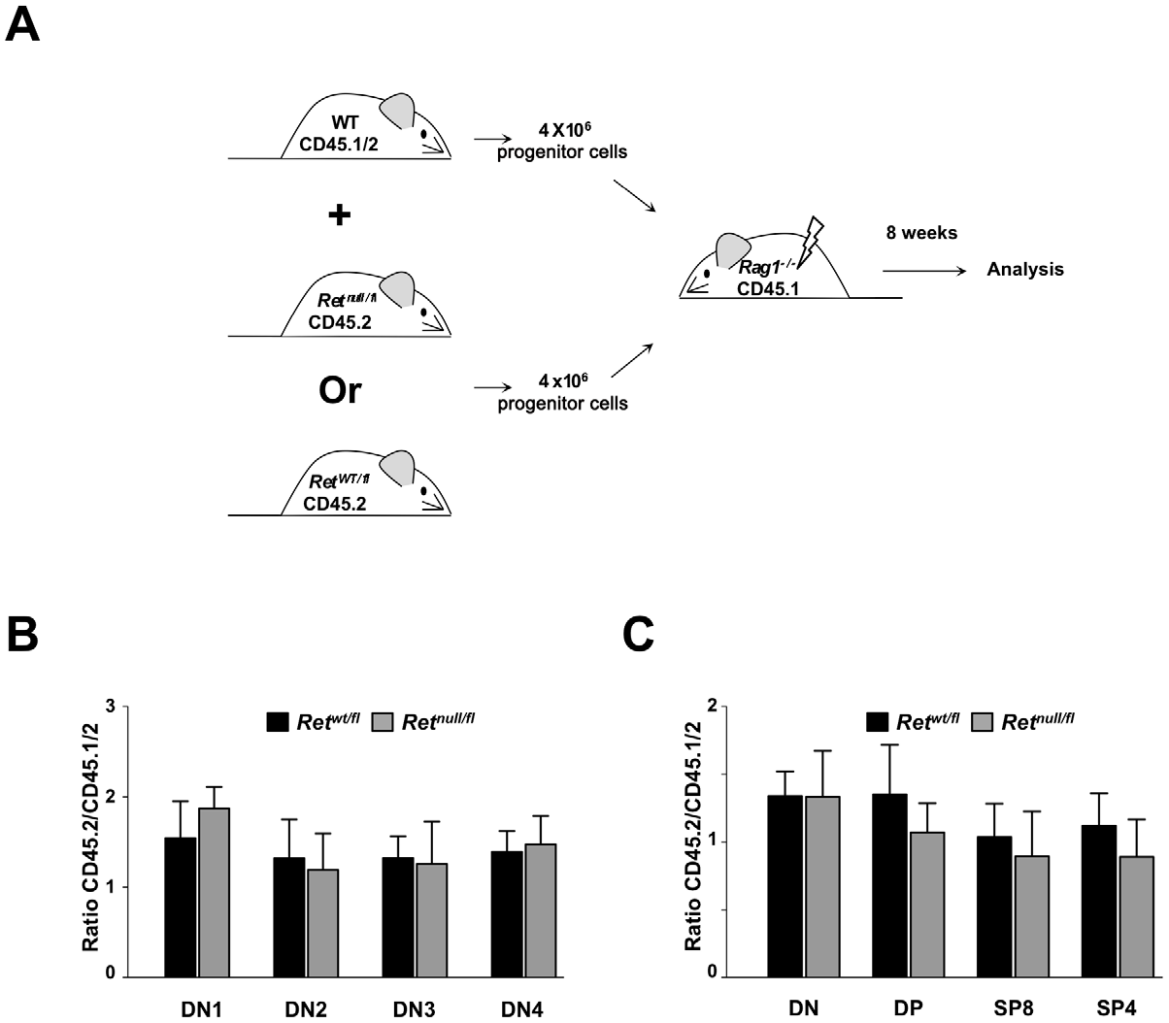
Competitive fitness and thymic reconstitution of *Ret-*null thymocytes. **A.** Experimental scheme: 9Gy irradiated hosts (*Rag1^−/−^*, CD45.1) received WT competitor precursors (CD45.1/2) together with *hCD2*Cre/*Ret^null/fl^* or control *hCD2*Cre^−^/*Ret^wt/fl^* precursors (CD45.2). **B.** 8 weeks after transplantation the thymus of the generated chimeras was analyzed by flow cytometry. Results show the ratio between *hCD2*Cre/*Ret^null/fl^* (grey bar) or *hCD2*Cre^−^/*Ret^wt/fl^* (black bar) and the third part WT competitor (CD45.1/2) through thymic T cell development. *hCD2*Cre/*Ret^null/fl^* precursor chimeras: n = 4; *hCD2*Cre^−^/*Ret^wt/fl^* precursor chimeras n = 4. Error bars show s.d. Two-tailed student *t*-tests were performed. No significant differences were found.

In order to specifically address that hypothesis, we performed sensitive competitive thymic repopulation assays to assess whether T cell progenitors lacking RET are able to compete with RET competent T cell progenitors. Bone marrow cells from *CD2*Cre/*Ret* ^null/fl^ or *CD2*Cre/*Ret* ^WT/fl^ (CD45.2) were transplanted into lethally irradiated *Rag1^−/−^* (CD45.1) recipients with C57Bl6 (CD45.1/2) competitor bone marrow cells in a 1∶1 ratio (Fig. 6A). 8 weeks after transplantation *Ret-*deficient (*CD2*Cre/*Ret* ^null/fl^) and *Ret*-competent (*CD2*Cre/*Ret* ^WT/fl^) thymocytes had similar fitness when compared to the third part competitor thymocytes (C57Bl6 (CD45.1/2)) across all analyzed stages of T cell development (DN1 to mature SP) (Fig. 6B, 6C). Thus, we conclude that RET signalling is dispensable for thymocyte competitive fitness and thymic reconstitution.

## Discussion

Our data indicate that the neuroregulatory genes *Ret*, *Gfra1* and *Gfra2* are expressed in discrete DN thymocytes, while *Gfra3* and *Gfra4* transcripts were absent in thymocytes. Interestingly, the RET ligands *Gdnf* and *Nrtn* are predominantly produced by non-hematopoietic thymic cells. These gene expression patterns raised the exciting possibility that RET signalling axes could control T cell development, adding to the growing body of evidence that the nervous and immune systems share similar key molecular signals [11], [14], [17], [18], [19], [25]. In line with this hypothesis, it was previously shown that GDNF could promote survival and maturation of thymocytes *in vitro* [11].

In order to test whether RET signalling axes control T cell development, we analyzed the thymus of genetically mutant embryos for *Ret*, *Gfra1* or *Gfra2* at E18.5 [20], [21], [22]. Despite expression of *Ret* in foetal thymocytes, *Ret* deficient embryos showed normal DN, immCD8 DP and γδ T cell development. These findings were also consistent with normal T cell development in null mice for the RET co-receptors that provide specificity to the neurotrophic factors GDNF and NRTN, respectively *Gfra1^−/−^* and *Gfra2^−/−^* mice. Thus, we conclude that RET and its signalling partners GFRα1 and GFRα2, are dispensable for foetal T cell development.

Since T cell development in adulthood employs additional molecular mechanisms to foetal thymopoiesis, we investigated whether *Ret* related genes controlled adult T cell development. Our data indicate co-expression of *Ret*, *Gfra1* and *Gfra2* in the early DN1 stage, and production of *Gdnf* and *Nrtn* in the adult thymic microenvironment. Both foetal and adult immature thymocytes co-express *Ret*, *Gfra1* and *Gfra2*. These data are in line with a previous report indicating expression of these genes in the foetal thymus by *in situ* hybridization [10]. However, DP thymocytes expressed the RET co-receptors *Gfra1* and *Gfra2*, despite absence of *Ret* expression, and similarly, adult DN2–4 expressed *Gfra1* but lacked significant *Ret* expression. This observation is in line with previous reports showing that GFRαs are more abundantly expressed than RET [26], [27], which suggests that GFRαs may modulate RET signalling in a non-cell-autonomous manner (signalling *in trans*) [28], [29]. Accordingly, we have recently shown that Lymphoid Tissue initiator cells use unconventional RET signalling in which receptor activation is provided by soluble ligand and co-receptors *in trans* secreted from nearby cells [17]. Thus, it is possible that RET negative, GFRα positive thymocytes may modulate the activity of neighbouring non-hematopoietic RET expressing cells.

Analysis of adult conditional *Ret* mutant mice (*Ret* ^fl/fl^) bred to CD2Cre mice revealed a significant, but small impact on the absolute numbers of DN1 thymocytes. However, this reduction was not translated into consecutive developmental stages, indicating that RET mediated signals are dispensable to T cell production *in vivo*. The co-expression of *Ret*/*Gfra1* in DN1 and the decreased number of DN1 cells in *Ret* ^fl/fl^ mice are consistent with a previous report indicating that GDNF promotes thymocyte survival in foetal thymic organ cultures (FTOCs) [11]. Nevertheless, our data does not support a major role for this signalling axis *in vivo*, since *Ret* mutant thymocytes develop normally and positive modulation of RET-signalling (*Ret* ^MEN2B^) was not beneficial for T cell development. Thus, the contribution of RET signalling to T cell development *in vivo* appears to be insignificant. Moreover, while FTOCs reproduce several aspects of T cell development [30], they fail to mimic the exact events in T cell development [31], [32], and therefore these different methodologies may also contribute to the observed discrepancies.

GDNF/GFRα1 have been shown to activate the transmembrane receptor RET and the neural cell adhesion molecule (NCAM) in neurons [33], [34]. Thus, although activation of a putative NCAM analogue by GDNF cannot be fully discarded in thymocytes, this is unlikely to have a significant physiological relevance since NCAM downstream signalling requires GFRα1 and *Gfra1* ^−/−^ embryos displayed normal thymopoiesis [33].

In order to overcome possible viability/proliferative compensatory mechanisms that may arise through T cell development, we performed sensitive competitive reconstitution assays *in vivo* with *Ret* deficient (*CD2*Cre/*Ret* ^null/fl^) and *Ret* competent (*CD2*Cre/*Ret* ^WT/fl^) thymocytes. Our data demonstrate that even in a very sensitive competitive setting the fitness of *Ret* deficient T cell precursors is intact.

Finally, our findings indicate that pharmacological inhibition of the RET pathway in severe pathologies, such as medullary thyroid cancer, should not be confronted with undesirable T cell production failure [15], [16].

In summary, our data demonstrate that RET signalling is dispensable to foetal and adult T cell development *in vivo*. Nevertheless, RET and its signalling partners are also expressed by mature T cells [19], thus, lineage targeted strategies will be critical to elucidate the contribution of RET signals to T cell function.

## Materials and Methods

### Mice

C57Bl/6J (CD45.2, CD45.1 and CD45.1/CD45.2), *Rag1^−/−^* (CD45.2 and CD45.1) [35], *CD2*Cre [23], *Gfra1^−/−^* [20], *Gfra2^−/−^* [21], *Ret^−/−^* [22], and *Ret* ^MEN2B^ [24] all in C57Bl/6J background, were bred and maintained at the IMM animal facility. All animal procedures were performed in accordance to national guidelines from the Direçao Geral de Veterinaria (permit number 420000000/2008) and approved by the committee on the ethics of animal experiments of the Instituto de Medicina Molecular.

### Generation of *Ret* conditional knockout mice

To generate mice harbouring a conditional *Ret* knock-out allele we engineered a targeting construct that firstly, included the introduction of a floxed 2.1 kb, Neomycin resistance (Neo^r^) cassette under the control of the phosphoglycerate kinase-1 (PGK) promoter and a polyA tail (pA). This cassette (PGK-NEO^r^-pA) was inserted approximately 4.5 kb upstream at the Xho I site of the pBluescript KS (pBS KS) vector that carried approximately 13 kb of the 5′ end of mouse *Ret* genomic locus flanking exon 1. The second modification included an insertion of a loxP ∼2.5 kb downstream of exon 1, at the Hind III site in the intron between exons 1 and 2 of the mouse *Ret* locus. Finally, a viral thymidine kinase cassette (∼3 kb) under the control of the PGK promoter (PGK-TK-pA) was inserted at the Hind III site ∼5 kb downstream of the inserted LoxP site. To obtain homologous recombination, this targeting construct was linearised by Xho I, purified by gel elution and extraction using the Qiaquick gel extraction kit (Qiagen), prior to electroporation into 129SvJ-derived R1 ES cells grown on mouse embryonic fibroblast (MEF) feeder layers. Following double selection with 300 µg/ml Geneticin (G418, Invitrogen) and 2 µM Gancyclovir (Sigma), positive clones were identified by Southern blotting. Genomic DNA was digested with Hind III restriction enzymes and a 5′ external probe of 500 bp was used to screen for positive clones. With the Hind III digest the WT and mutant alleles showed a band size of 16.5 kb and 6 kb respectively. Positive animals were subsequently crossed with transgenic mice expressing *Vav1*-iCre [23] in order to delete the PGK-NEO^r^-pA cassette. This recombination resulted in generating the floxed Ret mice wherein the two remaining LoxP sites were found flanking the first exon of the *Ret* locus, or the complete deletion of the first exon. These mice are further designated as *Ret* floxed (*Ret* ^fl^) and *Ret* null (*Ret* ^null^). Mice were further screened by PCR. Primer sequences were: P1: AAG CTC CCT CCT ACC GTG CT; P2: TGG GAT GAA CTC TGC CCA TT; P3: TGC TGC TCC ATA CAG ACA CA; P4: TAC ATG CTG TCT GCT CTC AG.

### Flow cytometry

Embryonic thymi were micro-dissected and either homogenized in 70 µM cell strainers or digested with Accutase medium (PAA Laboratories, Austria), 30′ at 37°. Adult thymi were homogenized in 70 µm cell strainers. Single cell suspensions were stained with the following antibodies from ebioscience, Biolegend, or BD: anti-CD3 (145-2C11), anti-CD4 (RM4-5), anti-CD8 (53-6.7), anti-CD44 (IM7), anti-CD25 (7D4), anti-CD45 (30-F11), anti-CD45.1 (A20), antiCD45.2 (104), anti-γδ TCR (GL3), anti-CD117 (2B8), anti-Sca1 (D7), Lineage (Lin) cocktail (anti-CD19 (eBio1D3), anti-CD11b (M1/70), anti-Gr.1 (RB6-8C5), anti-Ly79 (Ter119) and anti-NK1.1 (PK136)). Antibodies were coupled to FITC, PE, PerCP, PerCP-Cy5, PE-Cy7, APC, APC-Cy7, Pacific Blue, Brilliant Violet 421 and Horizon V500 fluorochromes or to biotin. Secondary incubation with fluorochrome binding streptavidin was performed when biotin coupled antibodies were used. Anti hRET was performed with antibody from R&D (132507) and respective anti-mouse IgG1 isotype control. Flow cytometry analysis was performed on a LSR Fortessa (BD) and data was analyzed with FlowJo 8.8.7 software (Tree Star). Cell-sorting was performed on a FACSAria I or FACSAria III (BD), and purity of obtained samples was >97%. CD45^+^ and CD45^−^ populations were sorted from the same samples.

### Real-time PCR analysis

RNA was extracted from sorted cell suspensions using RNeasy Micro Kit (Qiagen). RT-PCR was performed as previously described [18] and quantitative Real-time PCR for *Gfra1* and *Gfra2* were done as previously described [18], [36]. *Hprt1* was used as housekeeping gene. For TaqMan assays (Applied Biosystems) RNA was retro-transcribed using High Capacity RNA-to-cDNA Kit (Applied Biosystems), followed by a pre-amplification PCR using TaqMan PreAmp Master Mix (Applied Biosystems). TaqMan Gene Expression Master Mix (Applied Biosystems) was used in real-time quantitative PCR. TaqMan Gene Expression Assays bought from Applied Biosystems were: Gapdh Mm99999915_g1; *Hprt1* Mm00446968_m1; *Nrtn* Mm03024002_m1; *Gdnf* Mm00599849_m1; *Ret* Mm00436304_m1.

### Competitive reconstitution chimeras

Foetal livers from C57Bl/6 (CD45.1/CD45.2), *CD2*Cre/*Ret* ^null/fl^ (CD45.2, conditional knockouts) or *CD2*Cre/*Ret* ^WT/fl^ (CD45.2, controls) were made into single cell suspensions and enriched for precursors by staining with anti-CD117-APC followed by magnetic cell sorting with anti- APC microbeads (Miltenyi Biotec). Cells were then mixed in a 1∶1 ratio (50% CD45.1/CD45.2 and 50% CD45.2) and injected intra venous into irradiated (9Gy) *Rag1^−/−^* (CD451) hosts (4×10^6^ progenitor cells/host). Chimeras were analyzed 8 weeks after reconstitution.

### Statistics

Statistical analysis was done using Prism. Variance was analyzed using F- test. Student's t-test was performed on homocedastic populations and student's t-test with Welch correction was applied on samples with different variances.

## Supporting Information

Figure S1 **Impact of** ***Ret*** **,** ***Gfra1*** **or** ***Gfra2*** **ablation in embryonic thymocytes.** E18.5 thymocytes were analyzed by flow cytometry. **A.** Top: CD44 and CD25 expression profiles within the CD45^+^Lin^neg^CD3^−^DN compartment for *Ret^−/−^*, *Gfra1^−/−^*, *Gfra2^−/−^* and respective WT littermate controls. Bottom: absolute numbers of DN1–DN4 in *Ret*, *Gfra1*and *Gfra2* deficient mice. **B.** Dot plots: CD4 and CD8 expression profiles within the CD45^+^Lin^neg^γδTCR^−^ compartment from an example of *Ret^−/−^*and respective WT littermate controls. Similar gates were used in results shown. Note that within SPCD4 and SPCD8 gates >90% of cells were CD3^−^ and are thus immature thymocytes. Results show percentage and absolute numbers of immature CD8^+^ thymocytes and absolute numbers of DN and DP thymocytes in *Ret*, *Gfra1*and *Gfra2* deficient mice. **C.** Absolute numbers of γδ TCR^+^ thymocytes in *Ret*, *Gfra1*and *Gfra2* deficient mice. In all panels: Null mice: open symbols; WT littermate controls: full symbols; Mean value: dash line. Two-tailed student *t*-test analysis was performed between knockouts and respective WT littermate controls. No statistically significant differences were found.
(TIF)

Figure S2 **Generation of** ***Ret*** **conditional knockout mice.** **A.** Adult (8 weeks old) DN, DP, single-positive CD8 (SP8) and single-positive CD4 (SP4) thymocytes were purified by flow cytometry. RT-PCR analysis was performed. **B.** (A) The floxed Neomycin cassette was inserted ∼4.5 kb upstream of exon 1 of mouse Ret locus, a third loxP (LoxP3) was introduced downstream of exon 1 and ∼5 kb downstream the PGK-TK-pA cassette was inserted to aid negative selection. Targeted events were identified by Southern analysis of either Hind III digests of genomic DNA using the 5′ external probe. (B) The floxed allele was identified by PCR and the primers P1/P2 were used to identify the loxP that remained after excision of the Neomycin cassette (PGK-Neo-PA), while the loxP3 was identified using primers P3/P4. The primer sequences are in the methods section. (C) To screen for the null allele, primers P1 and P4 were used. **C.** Genotyping results from a litter of mice obtained from a *cRet131WT/null×cRet131fl/fl* breeding. In the loxP sites PCRs, upper band corresponds to the sequence with the loxP site and the lower band to the WT sequence. **D.** In order to evaluate the activity of Cre recombinase driven by hCD*2*, we bred hCD2Cre-expressing animals to *Rosa26 eYFP* animals. Histograms show flow cytometry analysis of eYFP expression in DN1 to DN4 thymocytes.
(TIF)

Figure S3 **Impact of** ***Ret*** **ablation in adult thymic development.** 8 week old *Ret* conditional knockout *hCD2*Cre^/^ *Ret^null/fl^* and control *hCD2*Cre^−^/*Ret^wt/fl^* mice were analyzed by flow cytometry. Results show absolute numbers of DN1–DN4 (top) and DN to mature single positive (bottom) in *hCD2*Cre/*Ret^null/fl^* (open circle) and control *hCD2*Cre^−^/*Ret^wt/fl^* (full circle) mice. Mean value: dash line. All WT and conditional *Ret* knockout deficient pairs were compared using two-tailed student *t*-tests, and no significant differences were found except where noted. *p<0.05.
(TIF)

Figure S4 **Impact of** ***Ret*** **gain-of-function mutation** ***Ret*** **^MEN2B^ in adult thymic development.** 8 week old *Ret* ^MEN2B/MEN2B^ (*MEN2B*) and their WT littermate controls were analyzed by flow cytometry. Results show absolute numbers of DN1–DN4 (top) and DN to mature SP (bottom) in *MEN2B* (open squares) and WT control (full circle) mice. Mean value: dash line. Two-tailed student *t*-test analysis was performed between knockouts and respective controls. No statistically significant differences were found.
(TIF)
