# Supplementary figures and images for: RET/GFRα Signals Are Dispensable for Thymic T Cell Development In Vivo
Source: PLoS One. 2012 Dec 27;7(12):e52949. doi: 10.1371/journal.pone.0052949 (PMC3531415; doi:10.1371/journal.pone.0052949)

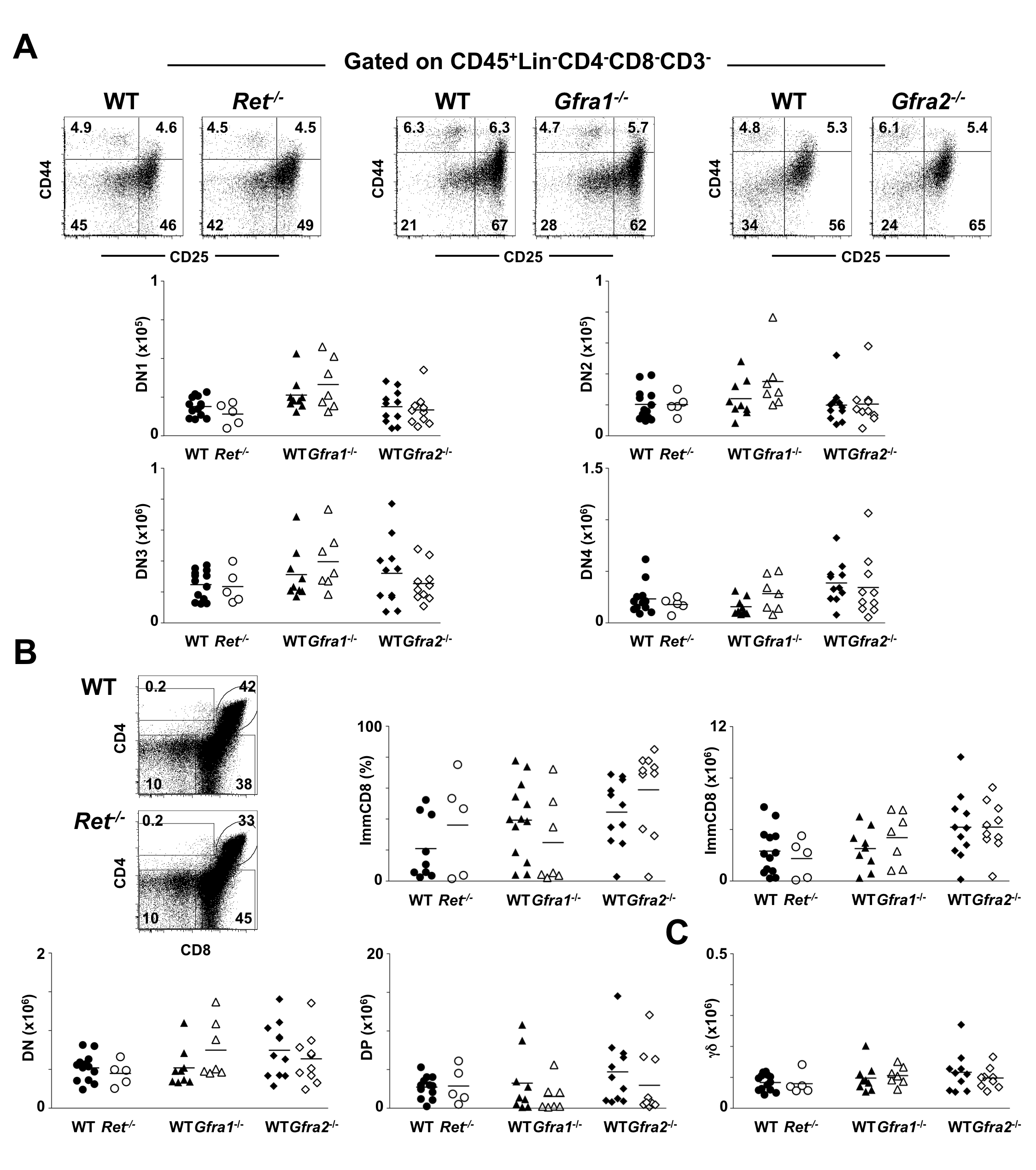

Supplement: Figure S1 — Impact of Ret , Gfra1 or Gfra2 ablation in embryonic thymocytes. E18.5 thymocytes were analyzed by flow cytometry. A. Top: CD44 and CD25 expression profiles within the CD45+LinnegCD3−DN compartment for Ret−/−, Gfra1−/−, Gfra2−/− and respective WT littermate controls. Bottom: absolute numbers of DN1–DN4 in Ret, Gfra1and Gfra2 deficient mice. B. Dot plots: CD4 and CD8 expression profiles within the CD45+LinnegγδTCR− compartment from an example of Ret−/−and respective WT littermate controls. Similar gates were used in results shown. Note that within SPCD4 and SPCD8 gates >90% of cells were CD3− and are thus immature thymocytes. Results show percentage and absolute numbers of immature CD8+ thymocytes and absolute numbers of DN and DP thymocytes in Ret, Gfra1and Gfra2 deficient mice. C. Absolute numbers of γδ TCR+ thymocytes in Ret, Gfra1and Gfra2 deficient mice. In all panels: Null mice: open symbols; WT littermate controls: full symbols; Mean value: dash line. Two-tailed student t-test analysis was performed between knockouts and respective WT littermate controls. No statistically significant differences were found. (TIF) [file pone.0052949.s001.tif]

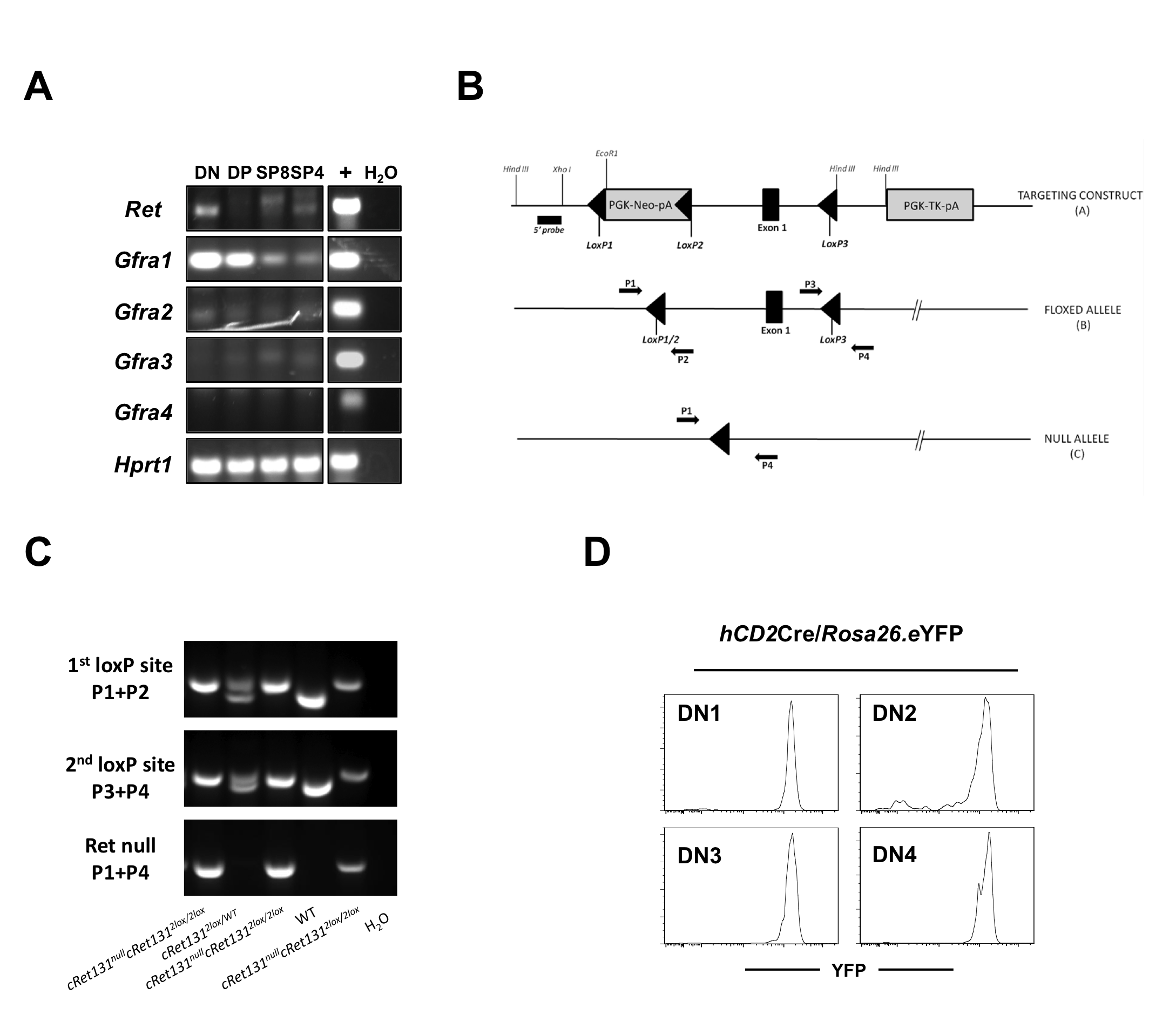

Supplement: Figure S2 — Generation of Ret conditional knockout mice. A. Adult (8 weeks old) DN, DP, single-positive CD8 (SP8) and single-positive CD4 (SP4) thymocytes were purified by flow cytometry. RT-PCR analysis was performed. B. (A) The floxed Neomycin cassette was inserted ∼4.5 kb upstream of exon 1 of mouse Ret locus, a third loxP (LoxP3) was introduced downstream of exon 1 and ∼5 kb downstream the PGK-TK-pA cassette was inserted to aid negative selection. Targeted events were identified by Southern analysis of either Hind III digests of genomic DNA using the 5′ external probe. (B) The floxed allele was identified by PCR and the primers P1/P2 were used to identify the loxP that remained after excision of the Neomycin cassette (PGK-Neo-PA), while the loxP3 was identified using primers P3/P4. The primer sequences are in the methods section. (C) To screen for the null allele, primers P1 and P4 were used. C. Genotyping results from a litter of mice obtained from a cRet131WT/null×cRet131fl/fl breeding. In the loxP sites PCRs, upper band corresponds to the sequence with the loxP site and the lower band to the WT sequence. D. In order to evaluate the activity of Cre recombinase driven by hCD2, we bred hCD2Cre-expressing animals to Rosa26 eYFP animals. Histograms show flow cytometry analysis of eYFP expression in DN1 to DN4 thymocytes. (TIF) [file pone.0052949.s002.tif]

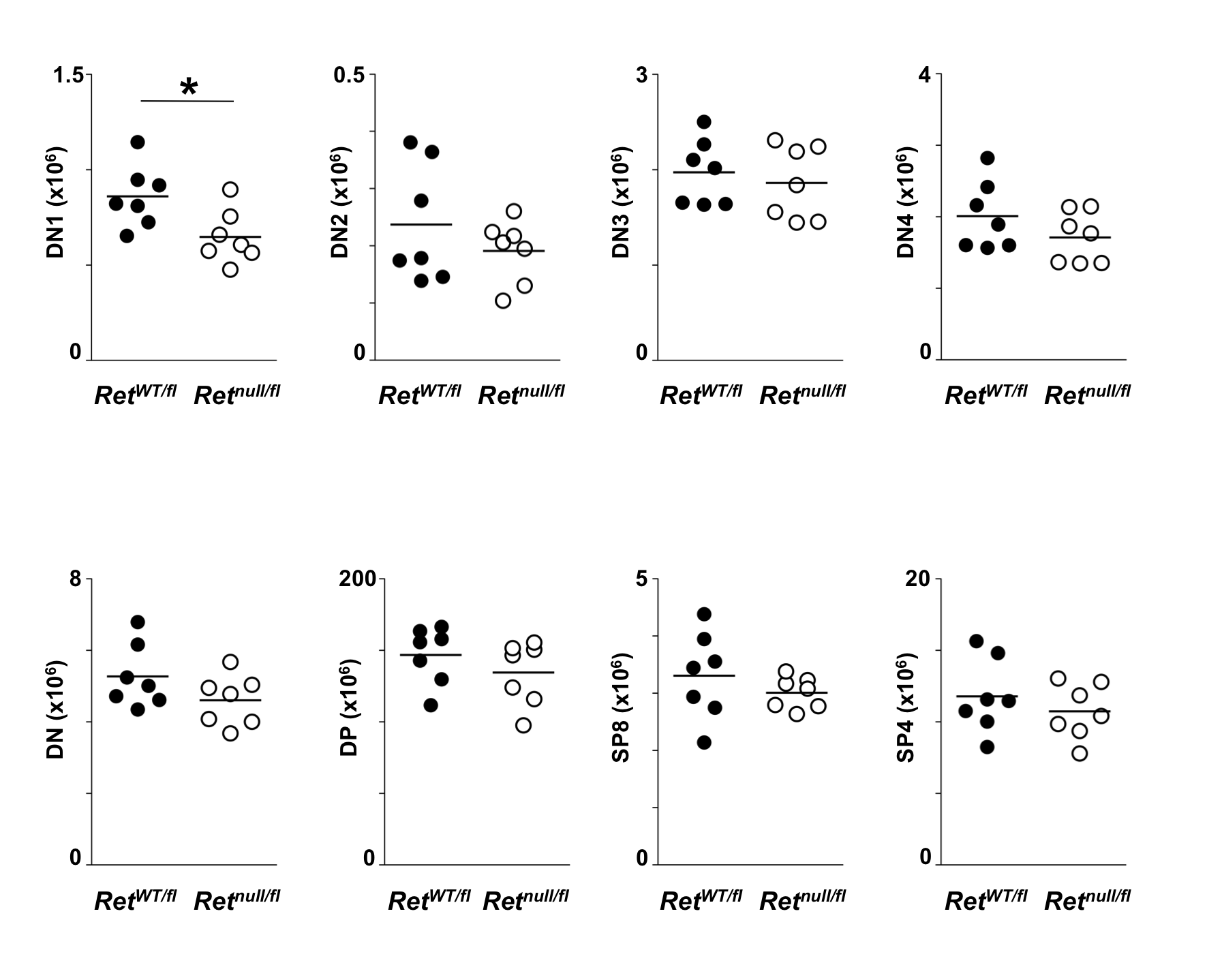

Supplement: Figure S3 — Impact of Ret ablation in adult thymic development. 8 week old Ret conditional knockout hCD2Cre/ Retnull/fl and control hCD2Cre−/Retwt/fl mice were analyzed by flow cytometry. Results show absolute numbers of DN1–DN4 (top) and DN to mature single positive (bottom) in hCD2Cre/Retnull/fl (open circle) and control hCD2Cre−/Retwt/fl (full circle) mice. Mean value: dash line. All WT and conditional Ret knockout deficient pairs were compared using two-tailed student t-tests, and no significant differences were found except where noted. *p<0.05. (TIF) [file pone.0052949.s003.tif]

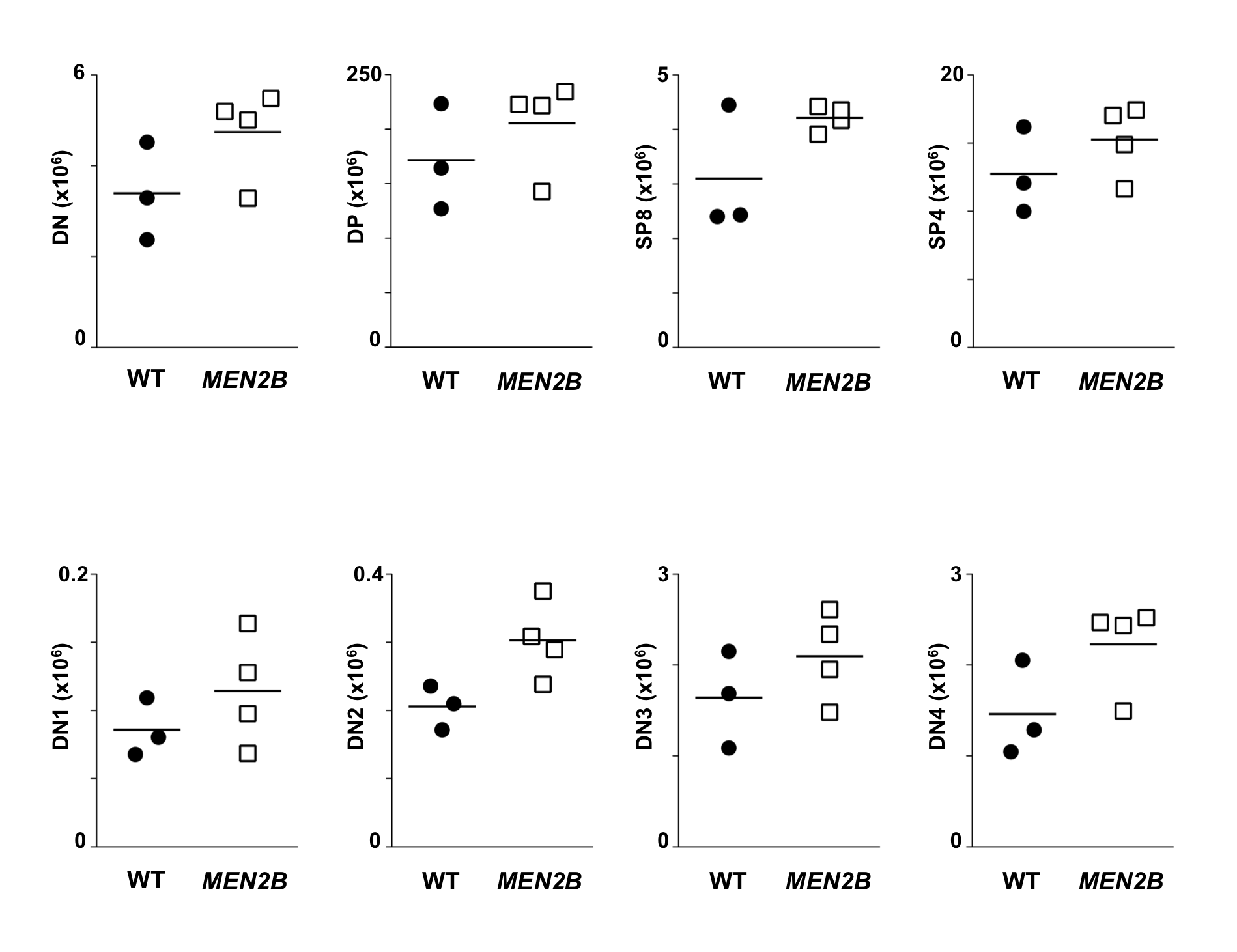

Supplement: Figure S4 — Impact of Ret gain-of-function mutation Ret MEN2B in adult thymic development. 8 week old Ret MEN2B/MEN2B (MEN2B) and their WT littermate controls were analyzed by flow cytometry. Results show absolute numbers of DN1–DN4 (top) and DN to mature SP (bottom) in MEN2B (open squares) and WT control (full circle) mice. Mean value: dash line. Two-tailed student t-test analysis was performed between knockouts and respective controls. No statistically significant differences were found. (TIF) [file pone.0052949.s004.tif]
